# An Innovative Sandwich Type Biosensor towards Sensitive and Selective Monitoring of 2-Arachidonoylglycerol in Human Plasma Samples Using P(*β*-CD)-AuNPs-DDT as Amplificant Agent: A New Immuno-Platform for the Recognition of Endocannabinoids in Real Samples

**DOI:** 10.3390/bios12100791

**Published:** 2022-09-26

**Authors:** Nastaran Aletaha, Kambiz Ghaseminasab, Mohammad Hasanzadeh, Fereshteh Kohansal, Yuqian Liu, Farzad Seidi

**Affiliations:** 1 Jiangsu Co-Innovation Center for Efficient Processing and Utilization of Forest Resources and International Innovation Center for Forest Chemicals and Materials, Nanjing Forestry University, Nanjing 210037, China; 2 Pharmaceutical Analysis Research Center, Tabriz University of Medical Sciences, Tabriz 5166/15731, Iran; 3 Food and Drug Safety Research Center, Tabriz University of Medical Sciences, Tabriz 5166/15731, Iran; 4 Nutrition Research Center, Tabriz University of Medical Sciences, Tabriz 5166/15731, Iran; 5 Biotechnology Research Center, Tabriz University of Medical Sciences, Tabriz 5166/15731, Iran

**Keywords:** 2-arachidonoylglycerol, sandwich-type immunosensor, gold nanoparticles, biomedical electroanalysis, biomedicine, polymeric interface

## Abstract

In this work, 2-AG was successfully detected in human plasma samples using a new sandwich-type electrochemical immune device based on poly-*β*-cyclodextrin P(*β*-CD) functionalized with AuNPs-DDT and toluidine blue. The P(*β*-CD) ensured the bioactivity and stability of the immobilized 2-AG antibody by providing a broad surface for the efficient immobilization of the biotinylated antibody. To complete the top section of the immunosensor (reporter), an HRP-conjugated antibody of 2-AG (secondary antibody (Ab2)) was attached to the surface of a glassy carbon electrode (GCE) modified by P(*β*-CD), as well as a primarily biotinylated antibody (Ab1). The biosensor fabrication process was monitored using field-emission scanning electron microscope (FE-SEM) and EDS methods. Using the differential pulse voltammetry technique, the immunosensor was utilized for detection of 2-AG in real samples. The suggested interface increased the surface area, which allowed for the immobilization of a large quantity of anti-2-AG antibody while also improving biocompatibility, stability, and electrical conductivity. Finally, the suggested immunosensor’s limit of quantitation was determined to be 0.0078 ng/L, with a linear range of 0.0078 to 1.0 ng/L. The results showed that the suggested bioassay can be utilized for diagnosis of 2-AG in clinical samples as a unique and ultrasensitive electrochemical biodevice.

## 1. Introduction

The endocannabinoid system (ECS) is one of the body’s most critical systems, since it regulates a wide range of metabolic and physiological processes in the body. Endocannabinoids, which are endogenous lipid-based retrograde neurotransmitters bound to cannabinoid receptors (CBRs) and enzymes that are synthesized in the central nervous system (CNS), peripheral nervous system (PNS), and brain making up the ECS [1,2,3]. The ECS is involved in a variety of immune system functions, as well as regulating cognitive and physiological processes such as pregnancy, irritability, ovulation, hunger, emotion, and cognition, moderating the medicinal effects of cannabis and its derivatives. Furthermore, this system has an important role in the pathophysiology of a variety of illnesses and traumas, including attention-deficit hyperactivity disorder [4], sleep deprivation-related issues [5], and spinal cord injury [6,7,8,9,10,11]. 2-arachidonoylglycerol (2-AG) is an endocannabinoid that is generated from the omega-6 fatty acid, arachidonic acid, and glycerol and is the major endogenous ligand for the cannabinoid-2 receptor. It has cannabinoid neuromodulatory properties [12] and is found at rather high levels in the central nervous system. 2-AG has a number of pharmacological effects, including immunomodulation, suppression of prostate and breast cancer, cell proliferation, and neuroprotection. 2-AG has a stimulating anti-inflammatory and pro-inflammatory characteristic [13].

To date, some research efforts have been made to monitor 2-AG, such as high-performance liquid chromatography/mass spectroscopy (HPLC-MS), liquid chromatography/mass spectroscopy (LC-MS), LC-MS/MS, and enzyme-linked immunosorbent assay (ELISA) [14,15,16,17,18]. Routine approaches, despite their relative sensitivity and specificity in identifying arachidonic glycerol, have some limitations such as the need for specialized equipment and expert operators, as indicated in Table S1 (see Supporting Information) [14,15,16,17,18].

However, these methods have some limitations, such as requiring a time-consuming process, having laborious sample preparation steps, being unsuitable for reusability, and being more expensive. Biosensors measure target molecules within a certain detection interval in a biological sample and are analytical measurement devices transforming this information into a meaningful electrical signal [19]. Because of the therapeutic importance of 2-AG, as well as its rapid degradation and enzymatic sensitivity, efficient approaches to its precise detection are highly demanded [20]. Therefore, novel methods are now being developed to overcome the limitations of traditional methods.

Electrochemical immunosensors have recently gained popularity due to their unique characteristics such as sensitivity, speed, and selectivity in analysis. Electrochemical sandwich-type immunosensors, on the other hand, are attracting a lot of attention because of their high sensitivity, easy fabrication, rapid/direct analysis, and suitable selectivity [21,22,23]. Recently, some of nanomaterials were used to increase the electrochemical signals of these biosensors towards increment of immunosensors sensitivity. Different nanomaterials have been utilized as labels for sandwich-type immunosensors, including metal nanoparticles, quantum dots, carbon materials, polymeric materials, and electroactive component-loaded nanoparticles.

*β*-Cyclodextrin (*β*-CD) has a unique structure that includes seven D-glucose units and a spiral shape [24,25]. It has a hydrophobic interior chamber and a hydrophilic outside, giving it strong molecular binding and supramolecular detection capabilities [26]. As a result, *β*-CD and certain molecules form a stable host−guest interaction quickly. Gold nanoparticles (AuNPs) are used in analytical and biological fields due to their superior conductivity and high surface-to-volume ratios, which enhance the dense loading of biomolecules such as antibody and increment of biosensor sensitivity [27,28]. In order to attach a secondary antibody to the electrode surface, glutaraldehyde (GA) plays an important role as a crosslinker [29]. Additionally, toluidine blue (TB), a tiny, stable, and cost-effective molecule, can be employed in electrochemical sensing as a redox indicator without the need for additional molecules such as H_2_O_2_ to produce electrochemical signals [30,31].

For the first time, a novel sandwich-type electrochemical immunosensor to determine 2-AG based on poly-beta-cyclodextrin (P(*β*-CD)), toluidine blue (TB), glutaraldehyde (GA), and gold nanoparticles-conjugated 1-dodecanethiol (AuNPs-DDT) was developed in this work. In our new design, the P(*β*-CD)-modified GCE was also used to trap primary antibodies (Ab1) in non-polar cavities due to their high stability and selectivity. AuNPs/GA/TB were applied to immobilize the secondary antibody (HPR-Ab2) to realize additional electrochemical signal amplification. The fabricated sandwich immunosensor’s electrochemical responsiveness was considerably improved. The experiment revealed an ultrasensitive performance for the detection of 2-AG in the real sample of plasma using the differential pulse voltammetry (DPV) technique. Our early research indicates that the described sandwich immunosensor can be utilized to detect 2-AG in real samples with high sensitivity.

## 2. Experimental

### 2.1. Chemicals and Reagents

Potassium ferrocyanide (K_4_Fe(CN)_6_∙3H_2_O), potassium ferricyanide (K_3_Fe(CN)_6_), potassium chloride (KCI), N-hydroxysuccinimide (NHS), 1-ethyl-3-(3 dimethylaminopropyl)carbodiimide (EDC), *β*-CD, bovine serum albumin (BSA), chloroauric acid trihydrate (HAuCl_4_)3H_2_O, glutaraldehyde (GA), 1-dodecanethiol (DTT), toluidine blue (TB), sodium borohydride, tetraoctylammonium bromide, ethanol, and toluene with a purity of 99.9% were all obtained from Sigma-Aldrich, Ontario, Canada. Fresh frozen plasma samples were obtained from Iran Blood Transfusion Research Center (Tabriz, Iran). Phosphate-buffered saline (PBS) solutions (0.05 M) with (pH = 4 (for polymerization) and 7.4 (for bioanalysis)) were prepared using Na_2_HPO_4_ (0.1 M) and NaH_2_PO_4_ (0.1 M) dissolved in deionized water. A 2-AG kit containing biotin-containing antibodies, a standard buffer, and various concentrations of standard antigen was purchased from ZellBio GmbH, (Berlin, Germany) by Padgin Teb Company (Tehran, Iran). Other reagents used in this study had an analytical degree.

### 2.2. Apparatus

The electrochemical experiments were assessed by a standard three-electrode cell (from Metrohm), containing an Ag/AgCl-saturated KCl as a reference electrode, the working electrode was a glassy carbon electrode (GCE) (d = 2 mm) (from Azar electrode Co., West Azerbaijan, Iran), and a Pt wire worked as a counter electrode. The assembly was powered by PalmSens electrochemical system with PSTrance 5.3 software as a running program (PS4.F1.05, Palm instruments, Utrecht, The Netherlands). In addition, in this study, in order to clean the glass carbon electrode surface (GCE) from organic and inorganic contaminations and electropolymerization of *β*-CD, the cyclic voltammetry (CV) technique was used. The other technique used for quantification was differential pulse voltammetry (DPV) with the following paramters: T_equilibration_, 2 s; E_begin_, −1.0 V; E_end_, 1.0 V; E_step_, 0.1 V; E_pulse_, 0.1 V; t_pulse_, 0.2 s; scan rate, 0.1 V/s. A field-emission scanning electron microscope (FE-SEM, Hitachi-Su8020, Hitachi High-Technologies, Praha, Czech) for analysis the morphology of the electrode surface was used. Moreover, the chemical elements on the modified electrode surface were analyzed using the energy-dispersive spectroscopy (EDS) of the MIRA3 TESCAN (Brno, the Czech Republic) model.

### 2.3. Electropolymerization of β-CD on the Surface of the GCE

The electro-polymerization of *β*-CD on the surface of the GCE was accomplished using the cyclic voltammetry technique, the electro-polymerization was carried out at potential from −2 to +2 V (40 consecutive cycles). Figure S1 (see Supporting Information) displays the cyclic voltammograms produced during the electropolymerization of *β*-CD process on the CGE. A cathodic peak (peak 1) was visible in the inset cycle at around 0.5 V. The anodic peaks appeared at 0.5 V (peak 2) and 1.5 V after the second cycle (peak 3). A new cathodic peak (peak 4) arose at −0.5 V as of the 7th cycle. The peak current rose in subsequent cycles, showing the formation of P(*β*-CD) on the surface of the CGE. In addition, the current grew quickly up until cycle 28 and then gradually lessened in strength in subsequent cycles, suggesting that the film may have achieved its largest thickness [32,33,34,35].

### 2.4. Synthesis of AuNPs-DDT

For this purpose, 500 µL of HAuCl_4_ (0.5 mM) were first dissolved in 100 mL of deionized water, stirred magnetically and heated to the boiling point under reflux conditions. At this stage, 5 mL of a sodium citrate solution (0.5 M) were quickly and step-by-step (10 step for each 500 µL) added to the above solution to change the yellow color of the solution to wine-red. The magnetic stirring of the mixture was continued, until the wine-red color remained constant. The above solution was stable at 4 °C for 2 months. 

For the synthesis of AuNPs-DDT, an aqueous solution of HAuCl_4_ (6 mL, 30 mM) was mixed with tetraoctylammonium bromide in toluene. The mixture was vigorously stirred, until the gold salt was completely transferred to the organic phase and 1-dodecanethiol (DDT) (34 mg) was added. Freshly, prepared aqueous sodium borohydride (5 mL, 0.4 mM) was added dropwise, and the solution was continuously stirred over a period of 3 h. The organic layer was subsequently extracted and evaporated to 2 mL. Ethanol (80 mL) was added to the organic layer and stored at −18 °C for 4 h, after which the black precipitate was filtered off and washed with ethanol (Merck Millipore, Darmstadt, Germany). The freshly made nanoparticles were subsequently suspended in toluene for further use.

### 2.5. Fabrication of the Electrochemical Immunosensor

**Figure 1:**
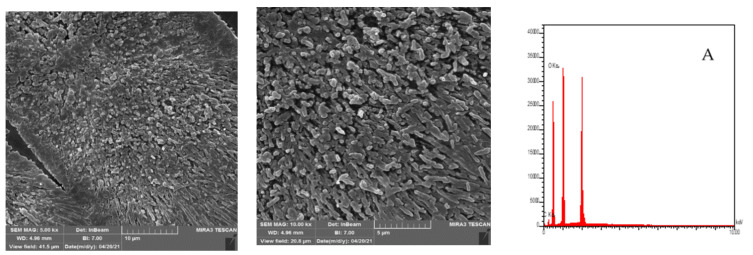

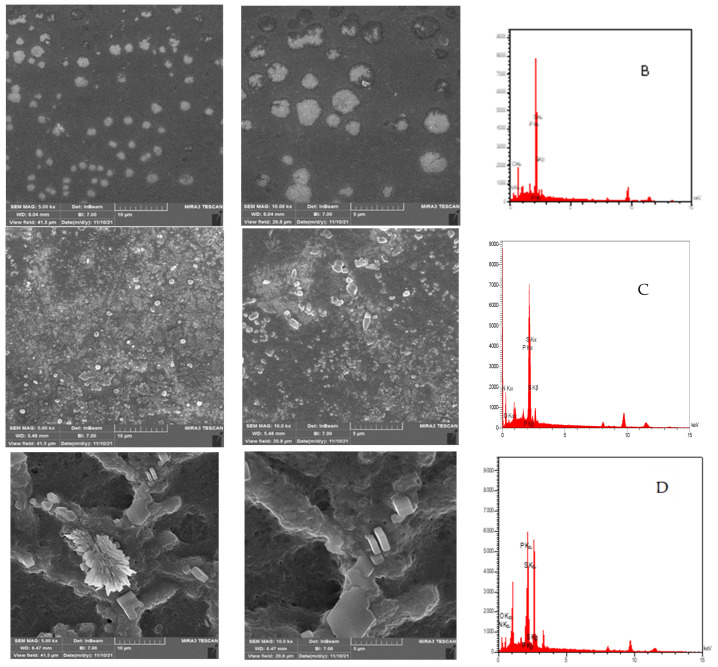
FE-SEM images of the P(*β*-CD)-modified GCE (**A**), the P(*β*-CD)-Ab1-modified GCE (**B**), the P(*β*-CD)-Ab1-BSA-2-AG-modified GCE (**C**), and the P(*β*-CD)-Ab1-BSA-2-AG-Ab2-HRP/AuNPs/TB/GLU-modified GCE (**D**) at different magnifications along with EDSs.

**Scheme 1:**
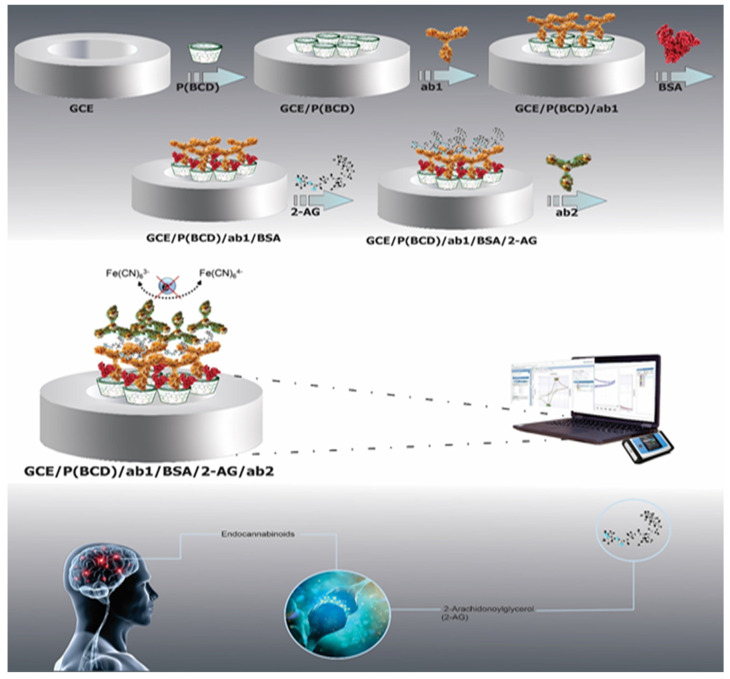
Fabrication of the electrochemical sandwich-type immunosensor for the sensitive detection of 2-AG.

Initially, the GCE was polished with 0.1 μm alumina slurry, until a mirror-like surface was obtained. Then, the electrode was electrochemically cleaned by the CV method, in the presence of a H_2_SO_4_ solution (0.1 M) in the potential range of −1.2 to 1.2 V using a scan speed of 0.1 V/s and more than 20 cycles. Afterwards, as previously shown in Figure 1, the polished GCE was exposed in a solution containing 6 mM *β*-CD in PBS (0.05 M) at pH = 4 for the electropolymerization of *β*-CD. The oxidation peaks in the range of 0.5 and 1.5 V in this CV graph indicated the fact that the *β*-CD was polymerized on the GCE surface successfully. To activate Ab1, 10 μL of EDC/NHS were combined with 5 μL of Ab1 (volume ratio: 2:1), and it was incubated at 4 °C for 30 min. After that, 5 μL of the above compound were incubated on the GCE at 4 °C for 4 h. Consequently, the prepared electrode surface was washed with a buffer washing solution to prevent any possible contamination. To block antibody-free areas, 10 μL of 10% BSA were dripped on the GCE electrode and then incubated for 30 min. At this stage, 5 μL of the 2-AG antigen were added to the electrode surface and incubated in the refrigerator at 4 °C for 2 h. The resulting electrode was washed with a washing buffer solution to remove infinite 2-AG molecules. Subsequently, 5 μL of the pre-prepared solution (HRP-Ab2/AuNPs/TB/GLU) were added to the surface of the 2-AG/Ab1/P(*β*-CD)-modified GCE at 4 °C for 2 h [35,36]. It is important to point out that the reporter part of immunosensor (HRP-Ab2/AuNPs/TB/GLU) was used for the signal amplification. Finally, the electrochemical properties of the sandwich-type immunosensor (Scheme 1) were evaluated by the CV and DPV techniques.

## 3. Results and Discussion

### 3.1. Characterization of AuNPs-DDT

#### 3.1.1. Investigation of the Morphology of the Synthesized AuNPs-DTT by FE-SEM

As can be seen in the Figure S2, the FE-SEM images showed that the AuNPs-DTT nanoparticles were in unique configurations with an average diameter of around 16 nm.

Energy-dispersive X-ray spectroscopy (EDS) is a chemical microanalytical technique usually performed in combination with SEM. X-ray photons have a certain energy that is specific to the components that created them. The EDS X-ray detector counts the X-rays that are released in accordance to their energy. A characteristic of the chemical elements that release X-rays is X-ray energy. As can be seen in the Figure S2, related to the nanocomposite (AuNPs-DTT), C, O, and Au had the maximum amounts in this step.

#### 3.1.2. Dynamic Light Scattering Analysis

The results obtained by dynamic light scattering (DLS) showed the average potential size of gold nanoparticles activated with DDT was about 29 nm (Figure S3A in Supporting Information). The size of the zeta potential gives an indication as to the colloidal system’s possible stability. If every particle in suspension has a significant negative or positive zeta potential, they will all have a tendency to repel one another and not have a tendency to clump together. The net surface charge of the nanoparticles when they are dissolved in a solution is known as zeta potential. The zeta potential, which has a significant impact on stability limitations, is either +30 mV or −30 mV. The zeta potential has to be either higher than +30 mV or lower than −30 mV for particles to be considered stable [35]. In this research, the AuNPs activated with DDT had a zeta potential of −66 mV, which showed that the particles were coated with DDT ions, causing a negative zeta potential (Figure S3B in Supporting Information).

As shown in Scheme 1, at first, 1 mL of the prepared AuNPs-DDT solution was mixed with 10 µL Ab2/HRP and stirred slightly. Subsequently, 20 µL glutaraldehyde were added as a crosslinker. Then, the mixture was refrigerated for 12 h (4 °C). At this stage, 1 mL of the obtained solution was mixed with 100 µL of TB (2 mM) as an electronic intermediate, and 20 µL of a BSA solution (1%) were added to block the non-specific adsorption sensor surface and to extinguish the possible remaining active points. Finally, it was washed with PBS and stored at 4 °C.

#### 3.1.3. Characterization of the Biosensor

Figure 1 shows the FE-SEM images of four modified GCEs at different magnifications at each stage of the immunosensor fabrication process. By generating holes on the surface of the GCE, *β*-CD was successfully polymerized, according to Figure 1A. Additionally, compared to the P(*β*-CD)-modified GCE (Figure 1B), the increase in surface roughness for the Ab1/P(*β*-CD)-modified GCE was a good indication that Ab1 uniformly covered the entire scan region. More crucially, after the incubation of the modified electrode with the 2-AG antigen (analyte), the shape of the electrode surface changed, indicating the formation of a linkage between Ab1 and 2-AG (Figure 1C). Interestingly, the FE-SEM images of HRP-Ab2/AuNPs/TB/GLU incubated on the modified electrode revealed a rougher surface, which led to an increase in surface active sites and, in turn, impacts the voltammetric measurement response sensitivity (Figure 1D). The EDS results also verified that the suggested immunosensor was successfully assembled.

More FESEM images are provided in Supporting Information (Figure S3).

### 3.2. Electrochemical Behavior of the Engineered Immunosensor

**Figure 2:**
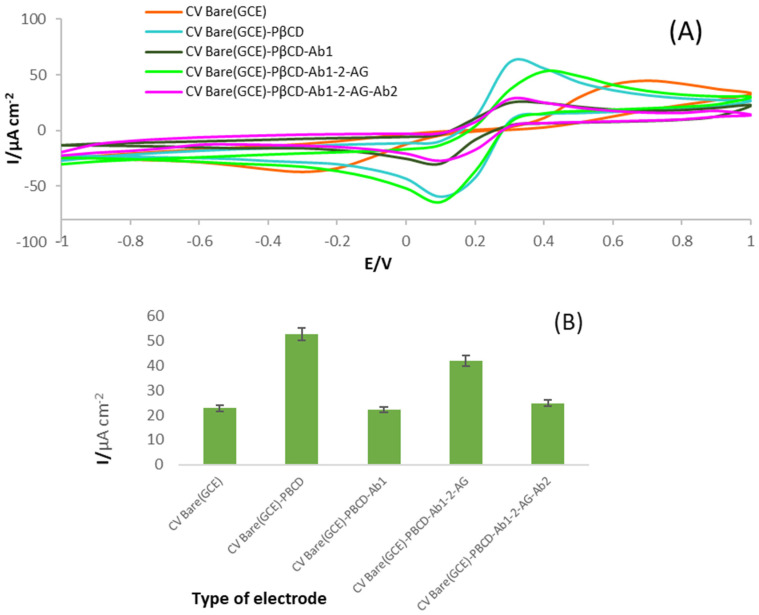
(**A**) CVs of the GCE, the P(*β*-CD)-modified GCE, the Ab1/P(*β*-CD)-modified GCE, the 2-AG/Ab1/P(*β*-CD)-modified GCE, and the HRP-Ab2/AuNPs/TB/GLU/2-AG/Ab1/P(*β*-CD)-modified GCE in a solution containing 0.03 M K_4_ Fe(CN)_6_/K_3_ Fe(CN)_6_/KCl in a potential range of −1 V to 1 V at a scan rate 0.1 V/s. (**B**) Histogram of the peak current versus the type of electrode.

The CV technique was used to evaluate the electrode modification in 0.03 M ferro/ferricyanide along with 0.01 M KCl, as shown in (Figure 2). Based on the obtained results, the characteristic peak current of the bare GCE was significantly increased after modification with P(*β*-CD), which could be related to its excellent properties, such as the high surface area and conductivity. Surprisingly, the results demonstrated that the incubation of Ab1 on the surface of the P(*β*-CD)-modified GCE changed the oxidation peak current of the redox probe (Fe(III)/Fe(IV)) significantly. The oxidation peak current of the redox probe using the P(*β*-CD)-modified GCE rose in proportion to the increase in effective surface area. Changes in current responses were clearly recognizable from CV and DPV graphs. The peak currents of redox probe were then reduced after incubating Ab1 on the surface of the GCE surface modified by P(*β*-CD). The insulation of the electrode surface by antibody (Ab1) as biological macromolecules reduced the efficiency of electron exchange from the mediator solution and delayed electron transfer kinetics. Therefore, the decrease of the peak currents after incubation in the capture antibody is due to the partially insulating barrier on the electrode surface produced by the presence of proteins. In addition, following 2-AG antigen incubation, the redox peak current of Fe(III)/Fe(IV) was lowered again compared to that at the previous stage, indicating that the antibody−antigen immunocomplex was successfully formed. This immunecomplex decreased conductivity and restricted the electron transport process. Following the incubation of Ab2-HRP, the electrochemical signals were lowered due to a hampered electron transfer effect in contrast to the P(*β*-CD)-modified GCE. However, its peak current was greater than that of the P(*β*-CD)/Ab1/(2-Ag)-modified GCE due to the advent of AuNPs beside the second Ab, which increased the conductivity of the electrode. 

### 3.3. Analytical Approach

**Figure 3:**
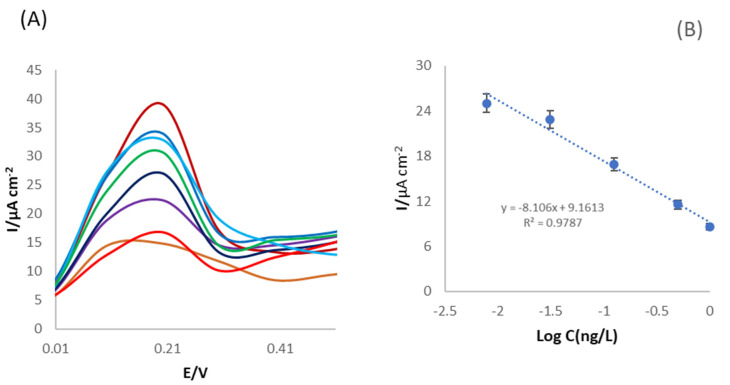
(**A**) DPV graphs of the engineered immunosensor at different concentrations of 2-AG (1, 0.25, 0.0625, 0.5, 0.125, 0.0312, 0.0078, and 0.00195 ng/L). The measurement conditions were similar to those shown as the legend of Figure 2. (**B**) Calibration curve (n = 3; SD = 1.77).

The electrochemical behaviors of the 2-AG immunosensor at various analyte concentrations were further examined using the DPV technique, which has a higher sensitivity and signal-to-noise ratio than CV. The immunosensor’s DPV responses for varied 2-AG concentrations under optimum circumstances in Fe(CN)_6_^3−/4−^ (0.03 M) containing KCl are shown in Figure 3, along with the accompanying calibration curve. According to the DPV data, well-defined peaks were created in the range of 0.0078 to 1 ng/L, which were proportionate to the concentrations of 2-AG. In addition, Figure 3B shows the calibration curve of the engineered immunosensor towards the determination of 2-Ag at different concentrations. From different concentrations (0.0078 to 1 ng/L), the linear regression equation was I_p_ (µA) = −8.106LogC (2-AG) + 9.1613 (R^2^ = 0.9787). The low limit of quantification (LLOQ) was determined to be 0.0078 ng/L (Figure 3B). In comparison to existing detection methods reported in analytical investigations (Table S1 in Supporting Information), the proposed immunosensor demonstrated improved or at least equal analytical performance in terms of the linear range and the detection limit. The following variables contributed to the outstanding sensing performance: (a) P(*β*-CD) had great conductivity and structural properties; (b) the fairly stable surface of its modified electrode provided a greater density and an advantageous area for focused antibody immobilization; (c) AuNPs-labeled HRP-Ab2 has been used as a substance for excellent catalytic reduction of ferro/ferricyanide with the support of the TB as a mediator to further augment the electrochemical signal. 

As described above and shown in Table S1 (see supporting information), while conventional methods (particularly chromatography-based techniques) revealed acceptable sensitivity, they are hard to operate, are expensive and need for expert personnel. The main purpose of this study is to eliminate the limitations and problems of routine methods in diagnosis of 2-AG. The results of the present study showed an LLOQ of 0.0078 ng/L and a liner range of 0.0078 to 1 ng/L, which are much better than previous studies.

### 3.4. Real Samples Analysis

The relevant biosensor and the suggested approach can be tested in real samples based on the acquired findings in the detection of 2-AG in standard samples. As a result, the DPV technique was used to record 2-AG concentrations in human plasma samples. The DPV technique was used to identify and measure the 2-AG biomarker in a human plasma sample. The DPV graphs (Figure S4A in Supporting Information) revealed that the designed immunosensor can detect the 2-AG biomarker in human plasma samples at concentrations ranging from 0.0156 to 0.5 ng/L, and the regression equation was as follows:

I (µA) = 11.113 × Log C(2-AG) + 24.139, R² = 0.9887.

The creation of immune complexes is linked to the corresponding peak, and the effect of the concentration may be measured reliably at various antigen concentrations. The calibration curve produced in the human plasma sample, on the other hand, revealed a linear relationship between the peak current intensity and the natural logarithm of the concentration. The method’s sensitivity was 11.113 µA/(ng/L). As a result, the designed immunosensor proved successful in detection and determination of 2-AG in real human samples.

### 3.5. Kinetic Study

The scan rate is one of the most important elements to consider when evaluating redox reactions for analyte detection. In addition, important information such as the electrochemical reaction process may be derived using the relationships between the peak current and the scan rate. The CV graphs of the P(*β*-CD)-modified GCE were recorded at varied sweep rates in the range of 10 to 900 mV/s in a 0.03 M ferro/ferricyanide solution to investigate the influence of the scan rate. The electro oxidation method was regulated by the diffusion reaction, as seen by the linear connection between the scan rate and the peak current (Figure S5 in Supporting Information). The influences of the sweep rate on the redox behavior, peak current, and electrode response were used to compute the unique surface coating of the GCE modified by P(*β*-CD). At low sweep rates, there was a linear relationship between the peak current and the sweep rate, as shown in (Figure S5A–C in Supporting Information), indicating the modifier’s catalytic nature. Furthermore, the developed interface (P(*β*-CD)-modified GCE) demonstrated a diffusion process for the probe utilizing the change of the peak current versus the square root of the sweep rate (Figure S5B in Supporting Information). Figure S5C (see supporting information) corroborated these findings. Controlled processes with absorption had a slop near 1.0, while controlled processes with diffusion had a gradient close to 0.5. The mass transfer of oxidation was regulated by the diffusion process, as shown in Figure S6C (see Supporting Information), since the chart’s slop is around 0.39. Figure S5D (see Supporting Information) also displays the Epa dependency on the Napierian logarithm of the sweep rate (ln*v*); the graph demonstrated that increasing the scan rate changed the peak potential, indicating that the redox behavior was irreversible.

### 3.6. Selectivity of the Immunosensor

For the analytical validation, the highly specific detection of 2-AG by the developed immunosensor was investigated. In this study, four antigens (i.e., prostate-specific antigen (PSA), carcinoembryonic antigen (CEA), CA15-3, and hyaluronic acid (HA)) were used in conjunction with 2-AG to determine the specificity of the designed biosensor by using the DPV technique (Figure S6A,C in Supporting Information). According to the results, the developed immunosensor had a high sensitivity for detecting 2-AG in the presence of interfering biomarkers (PSA, CEA, CA15-3, and HA). In other words, despite the presence of interfering biomarkers, this biodevice was capable of determining candidate analytes.

### 3.7. Repeatability of the Fabricated Substrate

To evaluate the repeatability between the electrodes, three electrodes polymerized by β-CD were simultaneously examined by recording CV graphs in a 0.03 M K_4_ Fe(CN)_6_/K_3_ Fe(CN)_6_/KCl solution. The results obtained in (Figure S7 in Supporting Information) confirmed the repeatability of the proposed substrate. 

### 3.8. Reproducibility of the Immunosensor

To evaluate the fabricated immunosensor reproducibility, three GCEs were modified under the same circumstances up to the P(*β*-DC)/Ab1-BSA/2-AG/Ab2 step. The computed avarage relative standard deviation was found to be 5.6%. According to Figure S8 (see supporting information), the GCE modified by P(*β*-DC)/Ab1-BSA/2-AG/Ab2 showed appropriate reproducibility [37,38].

### 3.9. Evaluation of the Stability

#### 3.9.1. Inter-Day Stability

**Figure 4:**
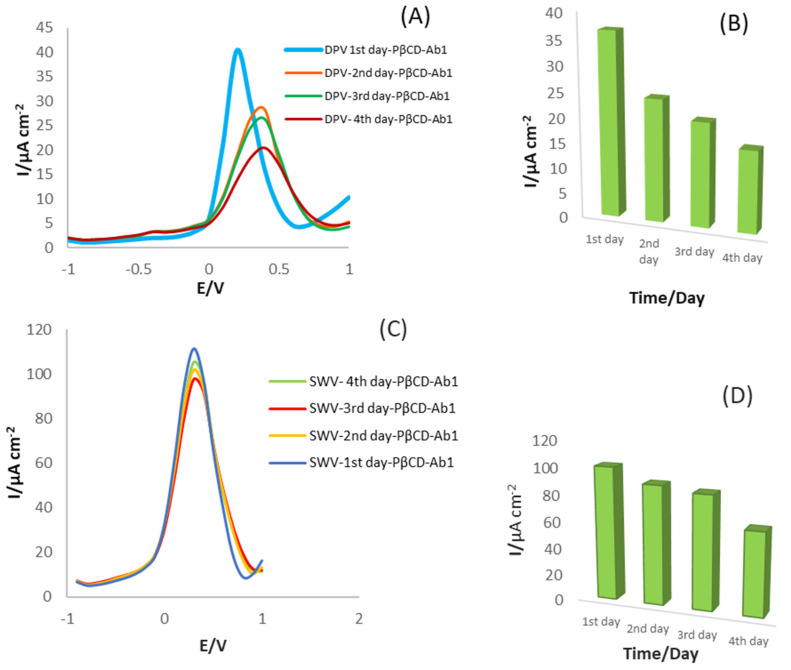
(**A**) DPV graphs of the P(*β*-DC)/Ab1-modified GCE at different storage times (1 to 4 days) in 0.03 M K_4_ Fe(CN)_6_/K_3_ Fe(CN)_6_/KCl as a supporting electrolyte with the potential range of −1 to +1 V at a scan rate of 0.1 V/s. (**B**) Histogram of the peak current versus the storage time of analysis (1–4 days) (n = 3, SD = 2.14). (**C**) SWV graphs of the P(*β*-DC)/Ab1-modified GCE at different storage times (1 to 4 days) in 0.03 M K_4_Fe(CN)_6_/K_3_Fe(CN)_6_/KCl as a supporting electrolyte with the potential range of −1 to +1 V at a scan rate of 0.1 V/s. (**D**) Histogram of the peak current versus the storage time of analysis (1–4 days) (n = 3, SD = 1.65).

One of the most significant aspects of the biosensor development is evaluating the stability of the proposed biosensors. For this purpose, the GCE was modified by P(*β*-CD)-Ab1. After four days, the long-term surface stability of the designed biosensor was assessed using SWV and DPV techniques (Figure 4). The stability of the biosensor developed on the first day was adequate, as shown in Figure 4. However, on the second, third, and fourth days, it displayed a significant decline. Both SWV and DPV techniques showed a diminishing trend in stability after four days, with this difference more noticeable in the DPV plot. The current intensities measured by DPV and SWV on the first day were 40 µA and 111 µA, respectively. Furthermore, on the second day, the Ip amount associated with DPV and SWV reduced to 28 µA and 101 µA, respectively. These values dropped to 26 and 97 µA on the third day, respectively, and finally on the fourth day, the current intensities decreased to 20 and 79 µA with the use of SWV and DPV, respectively. Perhaps the most important limitation of this immunosensor was its relatively low long-term stability. 

#### 3.9.2. Intra-Day Stability 

For this purpose, the stability of the engineered biosensor (P(*β*-CD)-Ab1-modified GCE) was investigated during a two-hour period. The created system demonstrated suitable stability after 2 h, according to DPV and SWV measurements (Figure S9 in Supporting Information). Thus, this immunosensor should be utilized immediately after fabrication.

#### 3.9.3. Cyclic Stability

The evaluation of polymeric substrate P(*β*-CD) stability by the CV technique for 1, 5, 10, 20, 50, 75, and 100 complete cycles in the potential range of −1 to 1 V and at a scanning rate of 100 mV/s was performed. the results showed that the substrate used to modify the surface of the GCE was stable, so that after 50 complete cycles, a slight change in the shape of the CV graph was observed (Figure S10 in Supporting Information).

## 4. Conclusions

In conclusion, an ultrasensitive and practical sandwich-type immunosensor for detecting 2-AG in human plasma samples was successfully established. To immobilize HRP-anti-2-AG, a novel nano-polymer of P(*β*-CD) was used as the supporting substrate, and a functionalized AuNPs/TB nanostructure was applied as the signal-amplifying probe. Using P(*β*-CD), toluidine blue, GLU, and gold nanoparticles-coupled AuNPs-DDT, a novel sandwich-type electrochemical immunosensor to detect 2-AG was created for the first time in this study. Because of its excellent stability and specificity, the P(*β*-CD)-modified GCE was also utilized in our innovative design to trap primary antibodies (Ab1) in non-polar canals. A secondary antibody (HPR-Ab2) was immobilized using AuNPs/GLU/TB to provide further electrochemical signal amplification. The electrochemical responsiveness of the constructed sandwich immunosensor was greatly enhanced. The suggested immunosensor had a broad linear range of 0.0078 to 1.0 ng/L under optimal operating conditions, with an LLOQ of 0.0078 ng/L. The immunosensor’s outstanding catalytic capabilities, improved surface area, favorable biocompatibility, strong electrical conductivity, and quick electron transfer may be responsible for its effective behavior. All of these characteristics demonstrated that the suggested immunosensor can be employed as a versatile platform for different biomarkers and has clinical and biological diagnostic potential.

## Data Availability

Not applicable.

## Supplementary Materials

The following supporting information can be downloaded at: https://www.mdpi.com/article/10.3390/bios12100791/s1, Figure S1: CV’s of electropolymerization of β-CD on the surface of the GCE in the presence of 6 mM of *β*-CD dissolved in 0.05M PBS (pH = 4) at 0.07 V·s^−1^ with 40 number of cycles; Figure S2: FE-SEM illustrations of AuNPs-DDT in different magnification along with EDS.; Figure S3: (**A**) DLS diagram of AuNPs-DDT. (**B**) Zeta-Potential of AuNPs-DDT.; Figure S4: (**A**) FE-SEM illustrations of P(*β*-CD)-GCE in different magnification along with EDS. (**B**): FE-SEM illustrations of P(*β*-CD)-Ab1-GCE in different magnification along with EDS. (**C**): FE-SEM illustrations of P(*β*-CD)-Ab1-BSA-2-AG GCE in different magnification along with EDS. (**D**): FE-SEM illustrations of P(*β*-CD)-Ab1-BSA-2-AG-Ab2-HRP/AuNPs/TB/GLU GCE in different magnification along with EDS.; Figure S5: (**A**) DPV responses of the immunosensor for different concentrations of 2-AG in human plasma sample: 0.5, 0.25, 0.125, 0.0625, 0.0312, 0.0156 ng/L in 0.03 M ferro/ferricyanide. (**B**) the calibrations curve. (n = 3, SD = 1.23); Figure S6: (**A**) CVs of P-βCD/GCE in the presence of 0.03M K_4_ Fe(CN)_6_ /K_3_ Fe(CN)_6_/KCl in different potential scan rates (10–900 mV/s) (**B**) Relationship between sweep rates and oxidation peak currents using CV technique. (**C**) Relationship between the Neperian logarithm of peak current (ln I_pa_) and Neperian logarithm of scan rate (ln *v*). (**D**) Variation of peak potential versus Neperian logarithm of sweep rates.; Figure S7: (**A**) DPVs of the designed immunosensor in the presence of (CEA, CA15-3, PSA, HA) in 0.03 M K_4_ Fe(CN)_6_ /K_3_ Fe(CN)_6_/KCl in a potential range of −1 V to 1V at scan rate of 0.1 V/s. (**B**) Histogram of peak currents versus the type of interfering agents (n = 3, SD = 1.95).; Figure S8: (**A**) CVs of three p(β-CD)/GCE in the potential range of −1 to 1 in the solution of 0.03 M K_4_Fe(CN)_6_ /K_3_ Fe(CN)_6_/KCl. (**B**) Histogram of peak currents vs. the number of electrodes.; Figure S9: (**A**) The DPVs of engineered immunosensor by three similar electrodes in the same condition. (**B**) Histogram pf peak current versus number of electrodes. Potential range of −1 to +1 V at a scan rate of 0.1 V/s.; Figure S10: (**A**) DPVs of P(*β*CD)/Ab1 modified GCE at different time of test. (**B**) Histogram of peak current versus storage time of test (n = 3, SD = 1.29). (**C**) SWVs of P(*β*CD)/Ab1 modified GCE at different time of test. (**D**) Histogram of peak current versus time of test (n = 3, SD = 2.04).; Figure S11: (**A**) CV of P(*β*-CD)-GCE (polymeric interface) in the potential range of −1 to 1 and scan rate of 0.1 V/s in 0.03 M of ferro/ferricyanide in different cycle numbers. (**B**) Histogram of peak current vs. number of cycles (n = 3, SD = 1.55); Table S1: Developed analytical methods for detection of 2-AG.
