# Supplementary material for: An Innovative Sandwich Type Biosensor towards Sensitive and Selective Monitoring of 2-Arachidonoylglycerol in Human Plasma Samples Using P(β-CD)-AuNPs-DDT as Amplificant Agent: A New Immuno-Platform for the Recognition of Endocannabinoids in Real Samples
Source: Biosensors (Basel). 2022 Sep 26;12(10):791. doi: 10.3390/bios12100791 (PMC9599568; doi:10.3390/bios12100791)
Supplement: Supplementary file 1 [file biosensors-12-00791-s001.zip › biosensors-1900243-supplementary.pdf]

Supporting Information

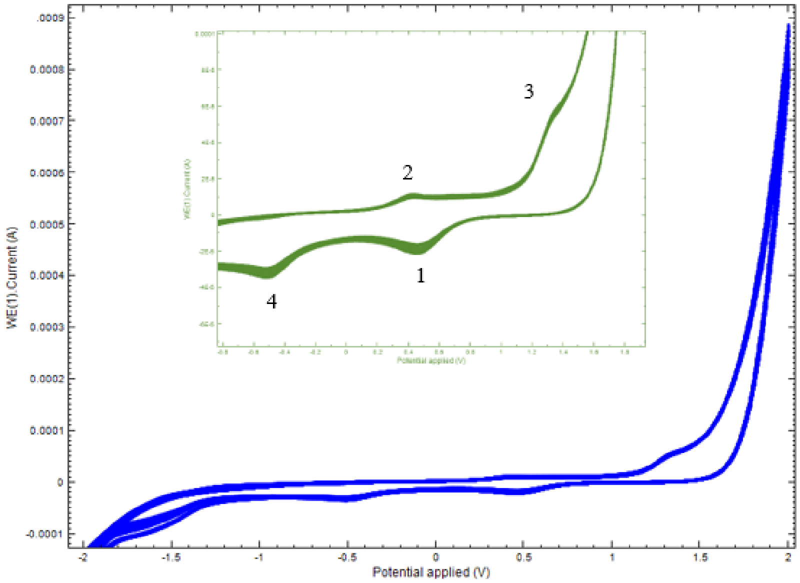

Figure S1. CV's of electropolymerization of β-CD on the surface of the GCE in the presence of 6 mM of β-CD dissolved in 0.05M PBS (pH=4) at 0.07 V.s^{-1} with 40 number of cycles**.**

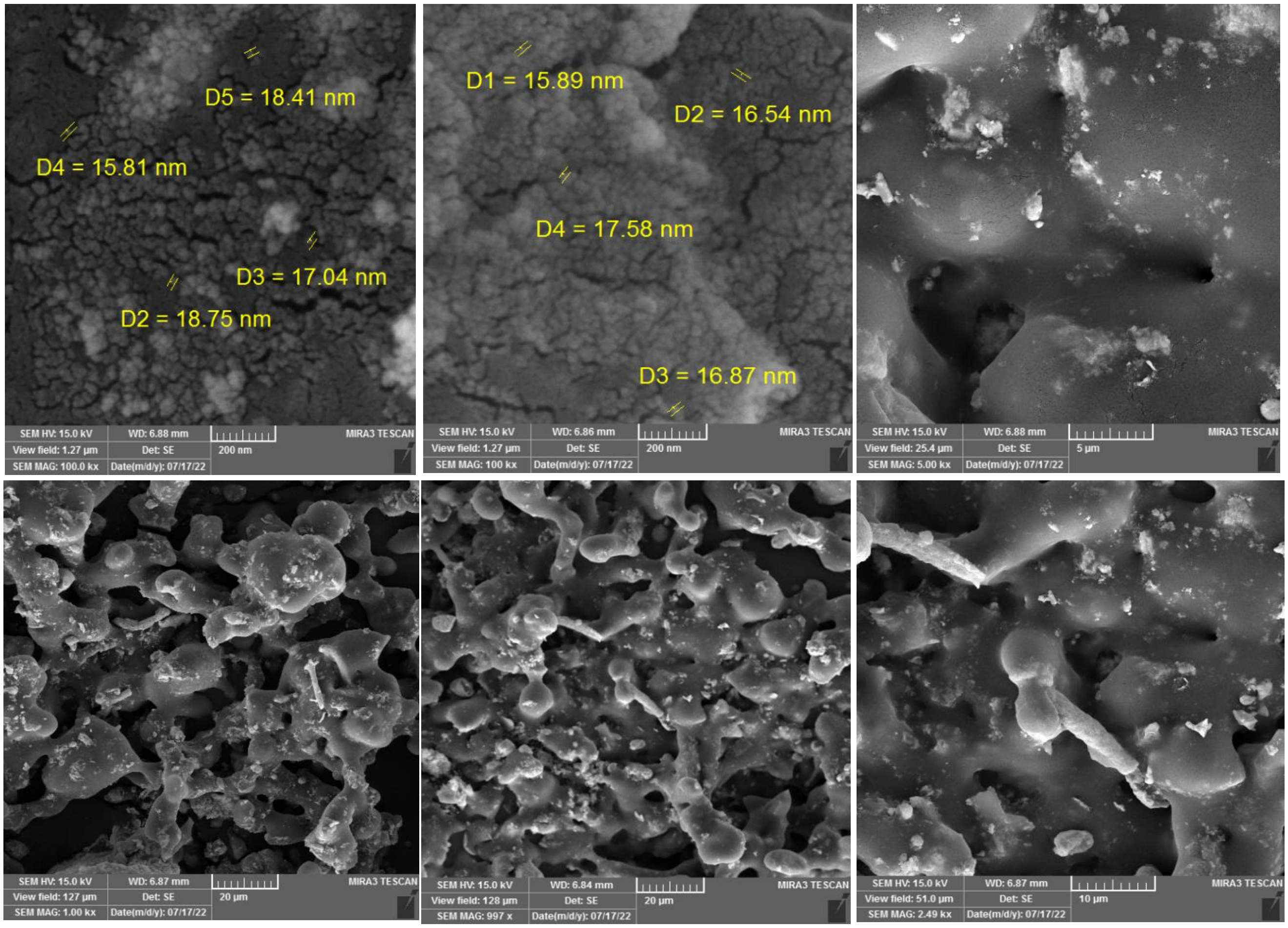

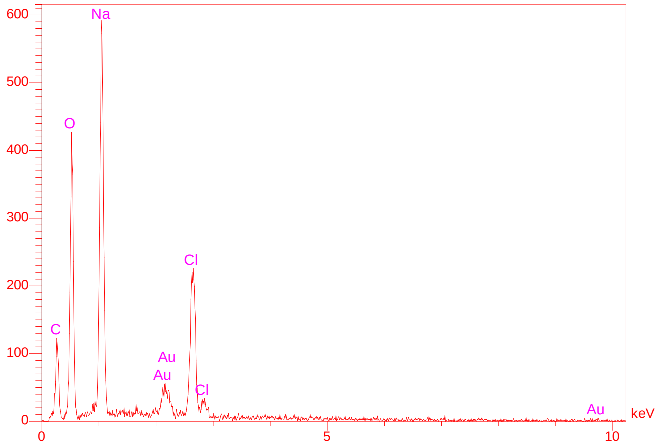

Elt	Line	Int	K	W%	A%
C	Ka	153.6	0.1385	23.50	37.47
O	Ka	545.8	0.2826	35.10	42.01
Na	Ka	889.2	0.2177	17.20	14.33
Cl	Ka	458.4	0.1622	8.68	4.69
Au	La	7.3	0.1990	15.51	1.51

Figure S2: FE-SEM illustrations of AuNPs-DDT in different magnification along with EDS.

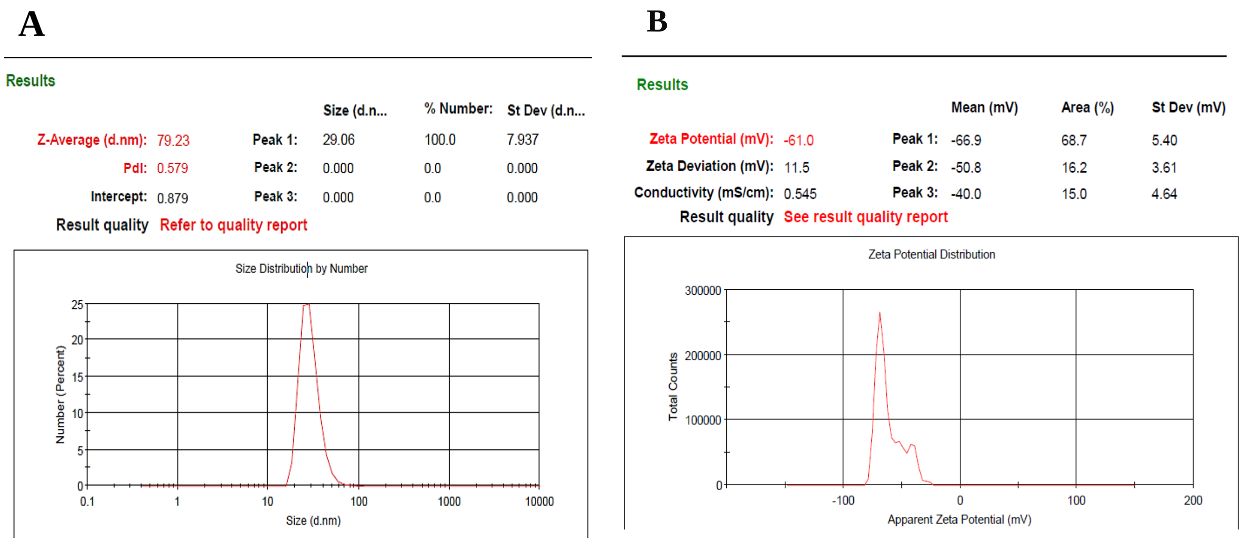

Figure S3. ***A)*** *DLS diagram of AuNPs-DDT.* ***B)*** *Zeta-Potential of AuNPs-DDT.*

A)

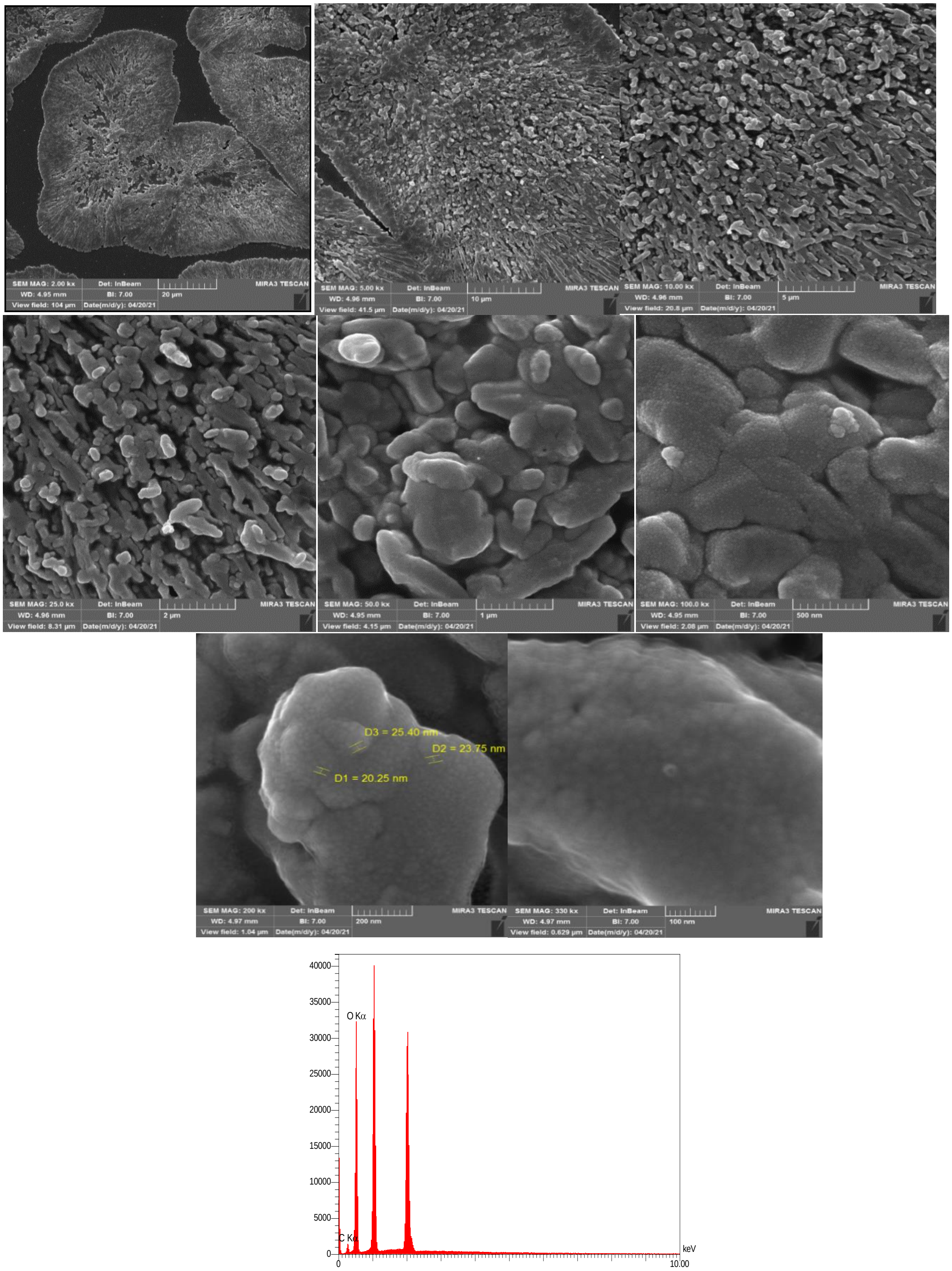

SEM MAG: 2.00 kx
Det: InBeam
WD: 4.95 mm
BI: 7.00
20 µm
View field: 104 µm
Date(m/d/y): 04/20/21
MIRA3 TESCAN
SEM MAG: 5.00 kx
WD: 4.96 mm
10 µm
View field: 41.5 µm
SEM MAG: 10.00 kx
5 µm
View field: 20.8 µm
SEM MAG: 25.0 kx
2 µm
View field: 8.31 µm
SEM MAG: 50.0 kx
1 µm
View field: 4.15 µm
SEM MAG: 100.0 kx
500 nm
View field: 2.08 µm
D3 = 25.40 nm
D2 = 23.75 nm
D1 = 20.25 nm
SEM MAG: 200 kx
WD: 4.97 mm
200 nm
View field: 1.04 µm
SEM MAG: 330 kx
100 nm
View field: 0.629 µm
40000
35000
30000
25000
20000
15000
10000
5000
0
O Kα
C Kα
keV
0
10.00

B.

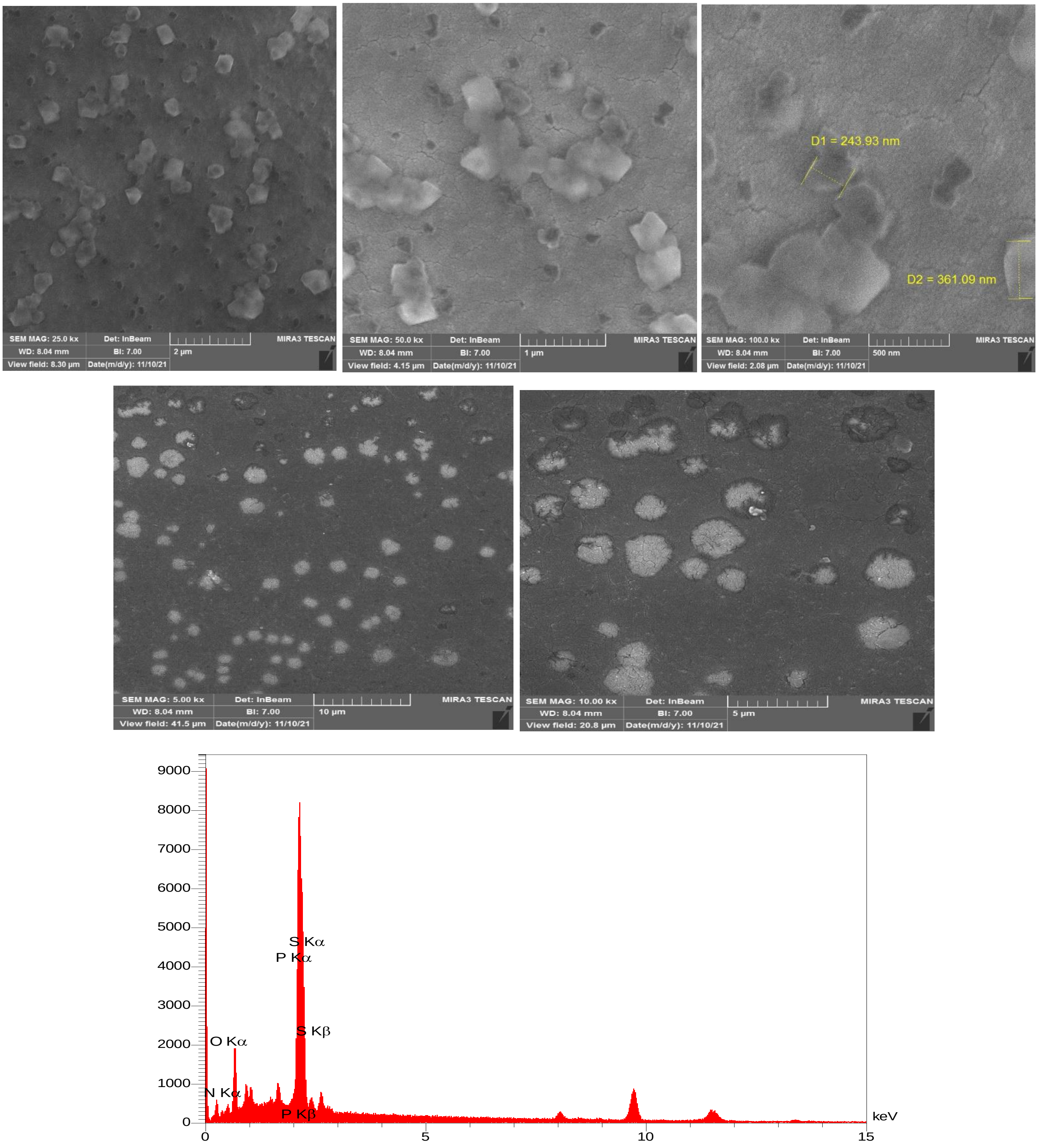

SEM MAG: 25.0 kx
Det: InBeam
WD: 8.04 mm
BI: 7.00
View field: 8.30 µm
Date(m/d/y): 11/10/21
2 µm
MIRA3 TESCAN
SEM MAG: 50.0 kx
Det: InBeam
WD: 8.04 mm
BI: 7.00
View field: 4.15 µm
Date(m/d/y): 11/10/21
1 µm
MIRA3 TESCAN
D1 = 243.93 nm
D2 = 361.09 nm
SEM MAG: 100.0 kx
Det: InBeam
WD: 8.04 mm
BI: 7.00
View field: 2.08 µm
Date(m/d/y): 11/10/21
500 nm
MIRA3 TESCAN
SEM MAG: 5.00 kx
Det: InBeam
WD: 8.04 mm
BI: 7.00
View field: 41.5 µm
Date(m/d/y): 11/10/21
10 µm
MIRA3 TESCAN
SEM MAG: 10.00 kx
Det: InBeam
WD: 8.04 mm
BI: 7.00
View field: 20.8 µm
Date(m/d/y): 11/10/21
5 µm
MIRA3 TESCAN
S Kα
P Kα
S Kβ
O Kα
N Kα
P Kβ
keV
0
5
10
15
1000
2000
3000
4000
5000
6000
7000
8000
9000

C.

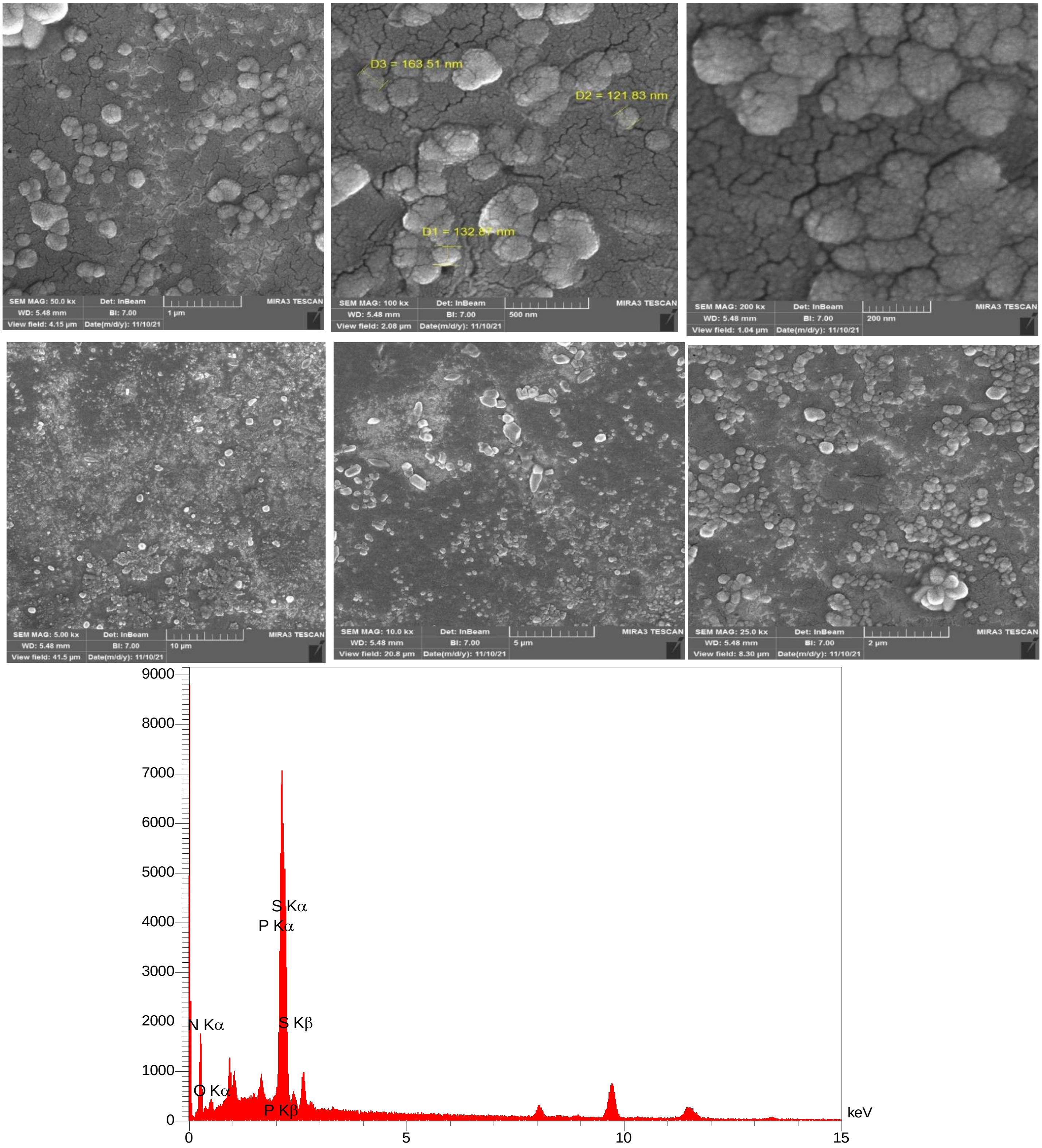

D3 = 163.51 nm
D2 = 121.83 nm
D1 = 132.87 nm
SEM MAG: 50.0 kx
SEM MAG: 100 kx
SEM MAG: 200 kx
SEM MAG: 5.00 kx
SEM MAG: 10.0 kx
SEM MAG: 25.0 kx
1 μm
500 nm
200 nm
10 μm
5 μm
2 μm
MIRA3 TESCAN
S Kα
P Kα
N Kα
S Kβ
O Kα
P Kβ
keV

D.

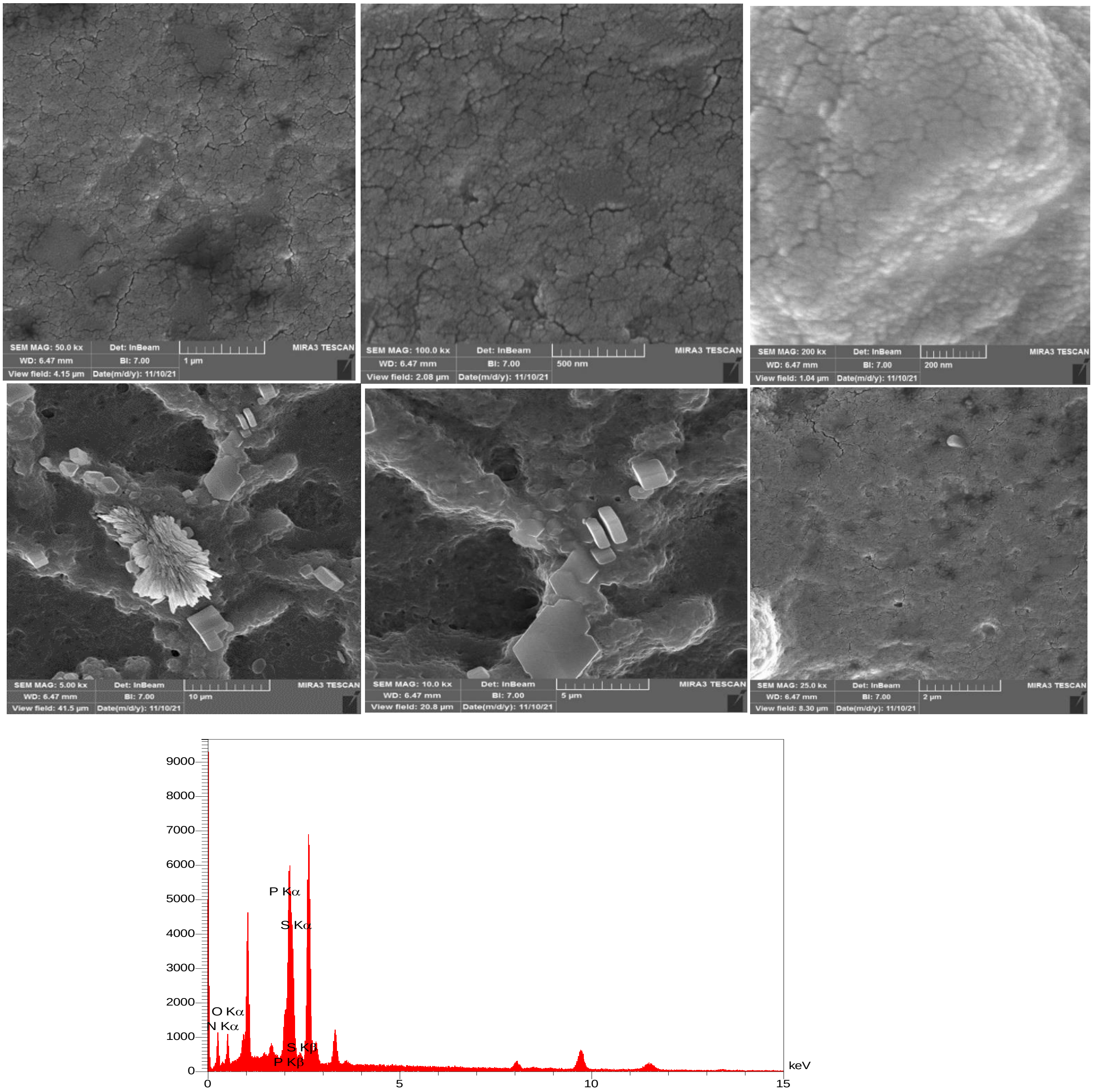

Figure S4. A: FE-SEM illustrations of P(β-CD)-GCE in different magnification along with EDS. **B:** FE-SEM illustrations of P(β-CD)-Ab1-GCE in different magnification along with EDS. **C:** FE-SEM illustrations of P(β-CD) -Ab1- BSA -2-AG GCE in different magnification along with EDS. **D:** FE-SEM illustrations of P(β-CD) -Ab1- BSA -2-AG- Ab2-HRP/AuNPs/TB/GLU GCE in different magnification along with EDS.

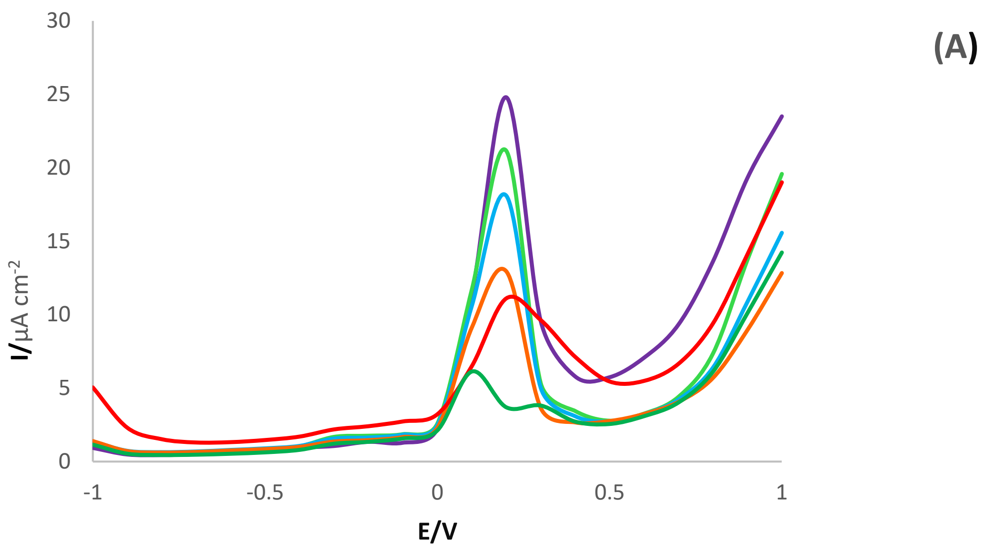

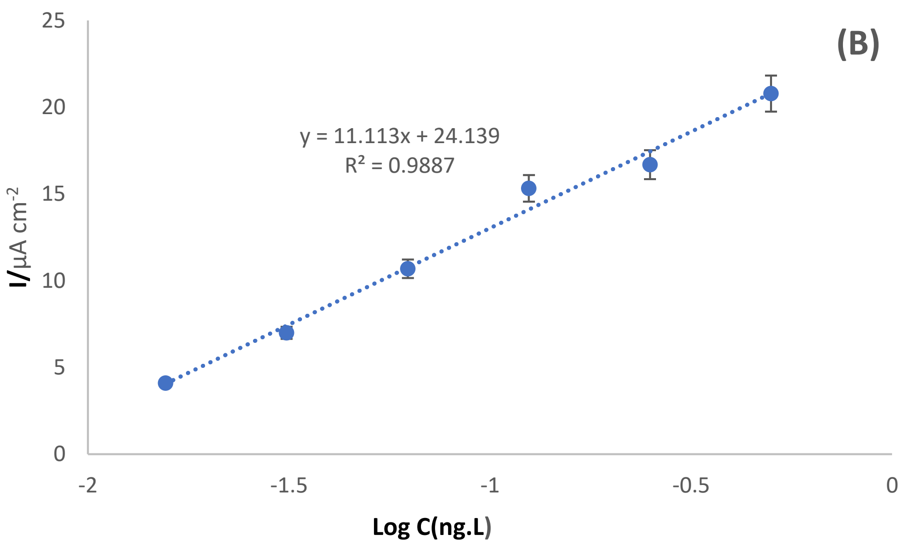

Figure S5. A) DPV responses of the immunosensor for different concentrations of 2-AG in human
plasma sample: 0.5, 0.25, 0.125, 0.0625, 0.0312, 0.0156 ng/l in 0.03M ferro/ferricyanide. **B)** the calibrations curves. (n=3, Sd=1.23).

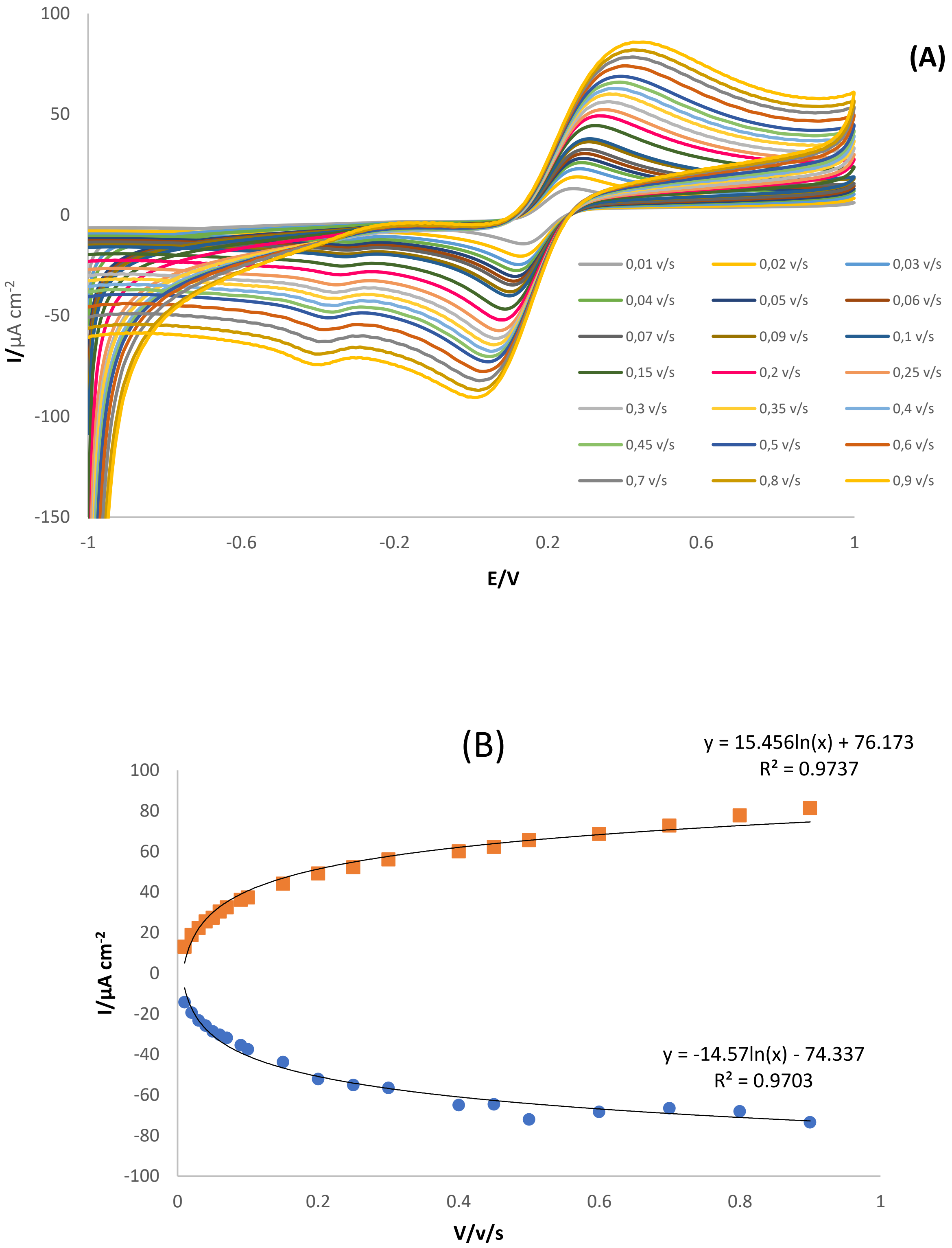
(A)
I/µA cm-2
E/V
100
50
0
-50
-100
-150
-1
-0.6
-0.2
0.2
0.6
1
0,01 v/s
0,02 v/s
0,03 v/s
0,04 v/s
0,05 v/s
0,06 v/s
0,07 v/s
0,09 v/s
0,1 v/s
0,15 v/s
0,2 v/s
0,25 v/s
0,3 v/s
0,35 v/s
0,4 v/s
0,45 v/s
0,5 v/s
0,6 v/s
0,7 v/s
0,8 v/s
0,9 v/s
(B)
y = 15.456ln(x) + 76.173
R² = 0.9737
y = -14.57ln(x) - 74.337
R² = 0.9703
I/µA cm-2
V/v/s
100
80
60
40
20
0
-20
-40
-60
-80
-100
0
0.2
0.4
0.6
0.8
1

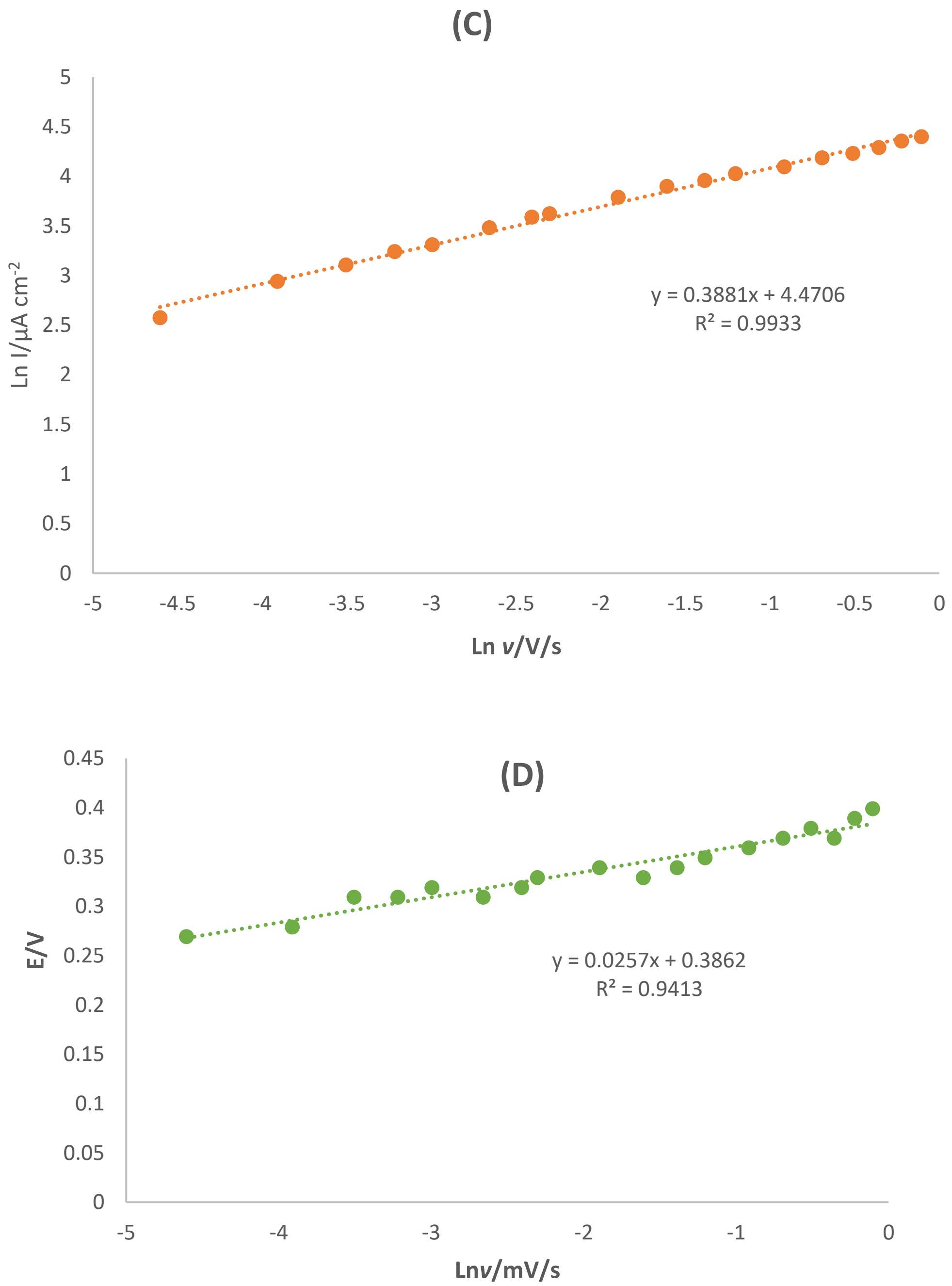

Figure S6. A) CVs of β-CD/GCE in the presence of 0.03M K_4 Fe(CN)$_6$ /K_3 Fe(CN)$_6$/KCl in different potential scan rates (10–900 mV/s) **B)** Relationship between sweep rates and oxidation peak currents using CV technique. **C)** Relationship between the Neperian logarithm of peak current (ln I_{pa}) and Neperian logarithm of scan rate (ln v). D) Variation of peak potential versus Neperian logarithm of sweep rates.

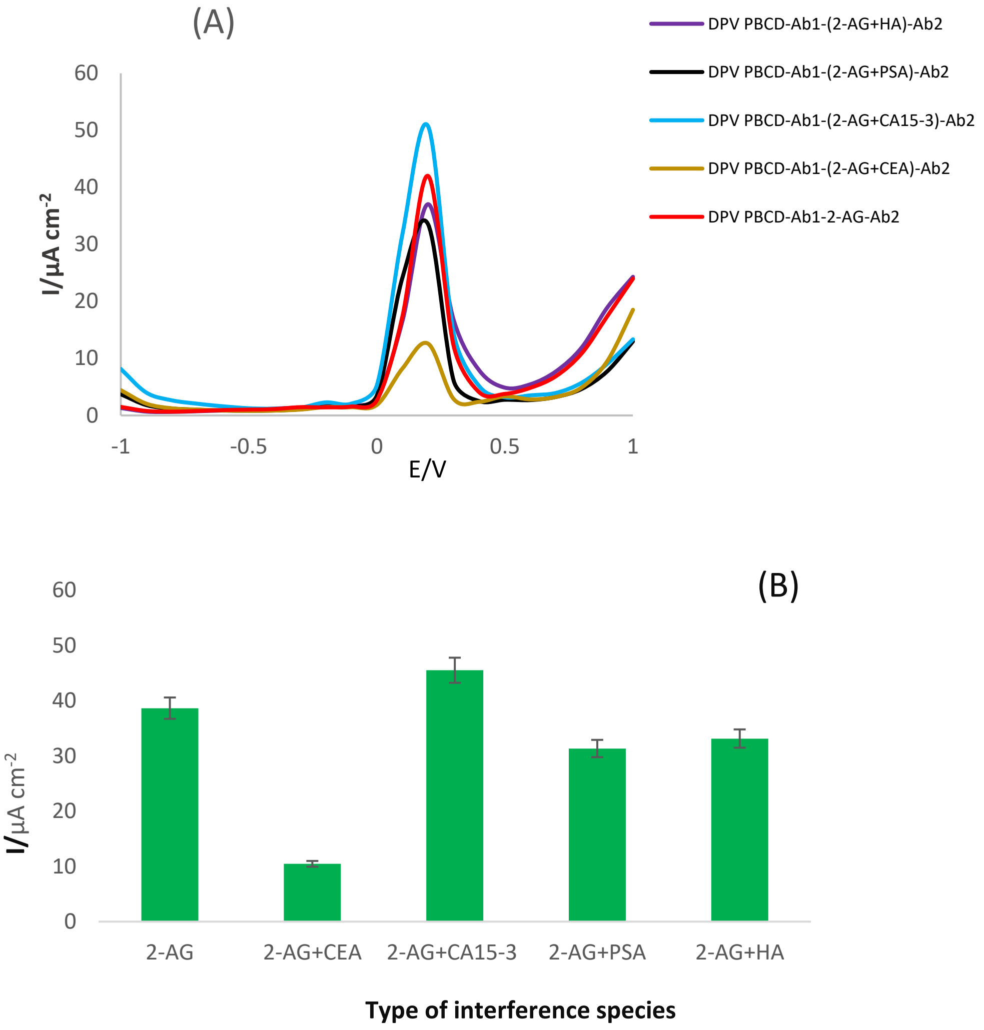

Figure S7. A) DPVs of the designed immunosensor in the presence of (CEA, CA15-3, PSA, HA) in
0.03 M K_4 Fe(CN)$_6$ /K_3 Fe(CN)$_6$/KCl in a potential range of -1 V to 1V at scan rate of 0.1 V/s. **B)** Histogram of peak currents *versus* the type of interfering agents (n=3, SD=1.95).

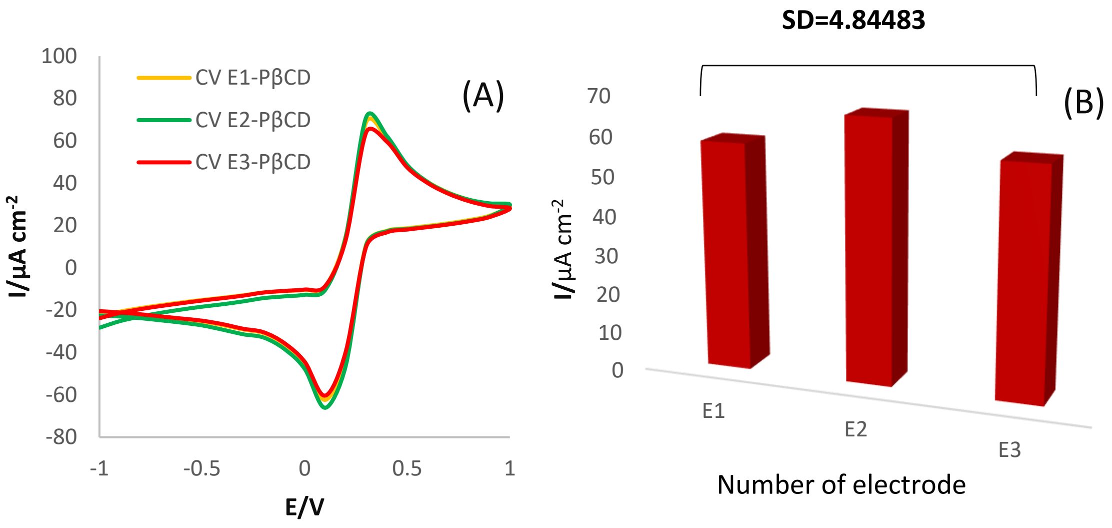

Figure S8. A) CVs of three p(β-CD)/GCE in the potential range of -1 to 1 in the solution of 0.03 M
$K_4Fe(CN)_6$ /K_3 $Fe(CN)_6$/KCl. **B)** Histogram of peak currents *vs*. the number of electrodes.

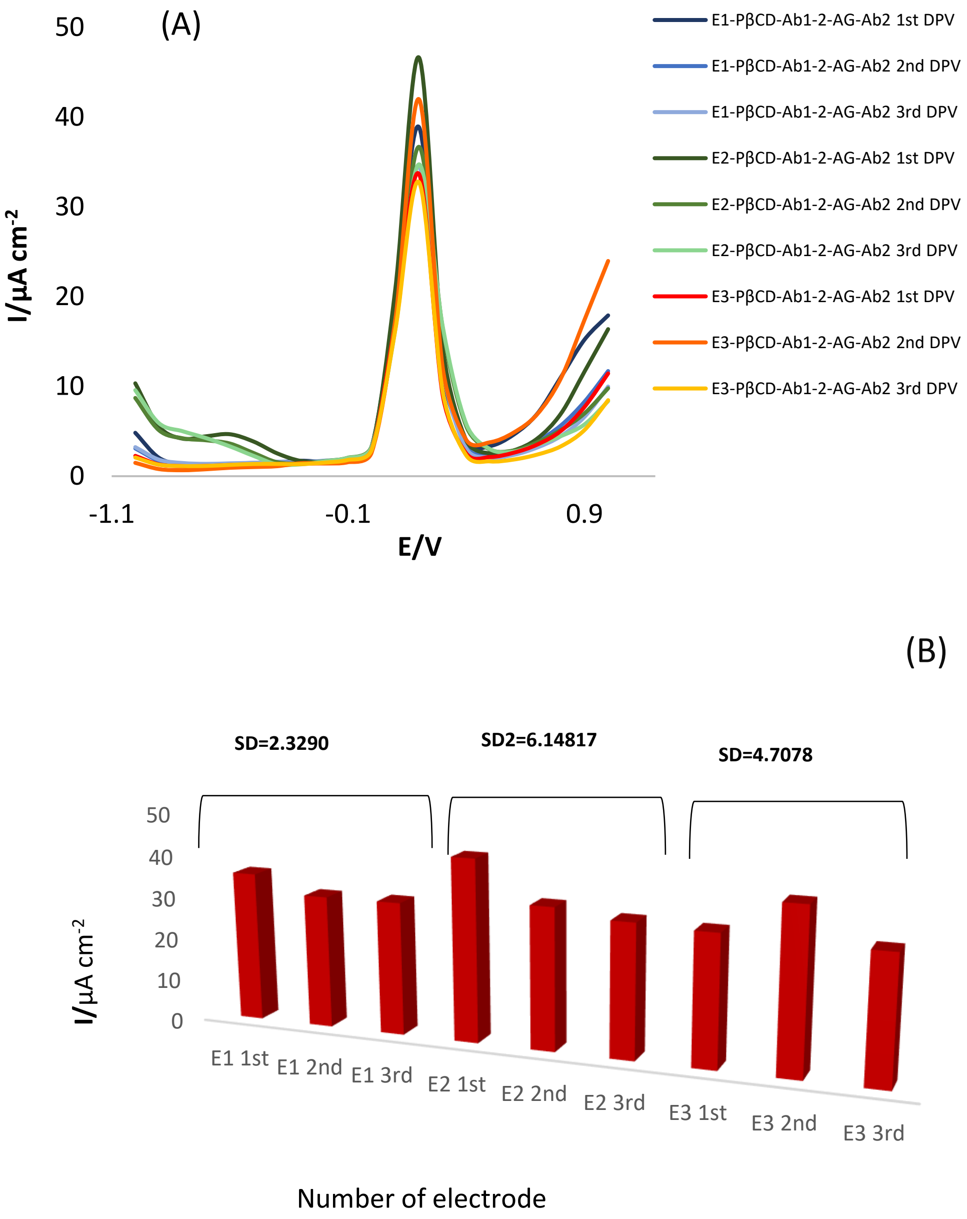

Figure S9. A) The DPVs of engineered immunosensor by three similar electrodes in the same condition. **B)** Histogram pf peak current versus number of electrodes. Potential range of -1 to +1 V at a scan rate of 0.1 V/s.

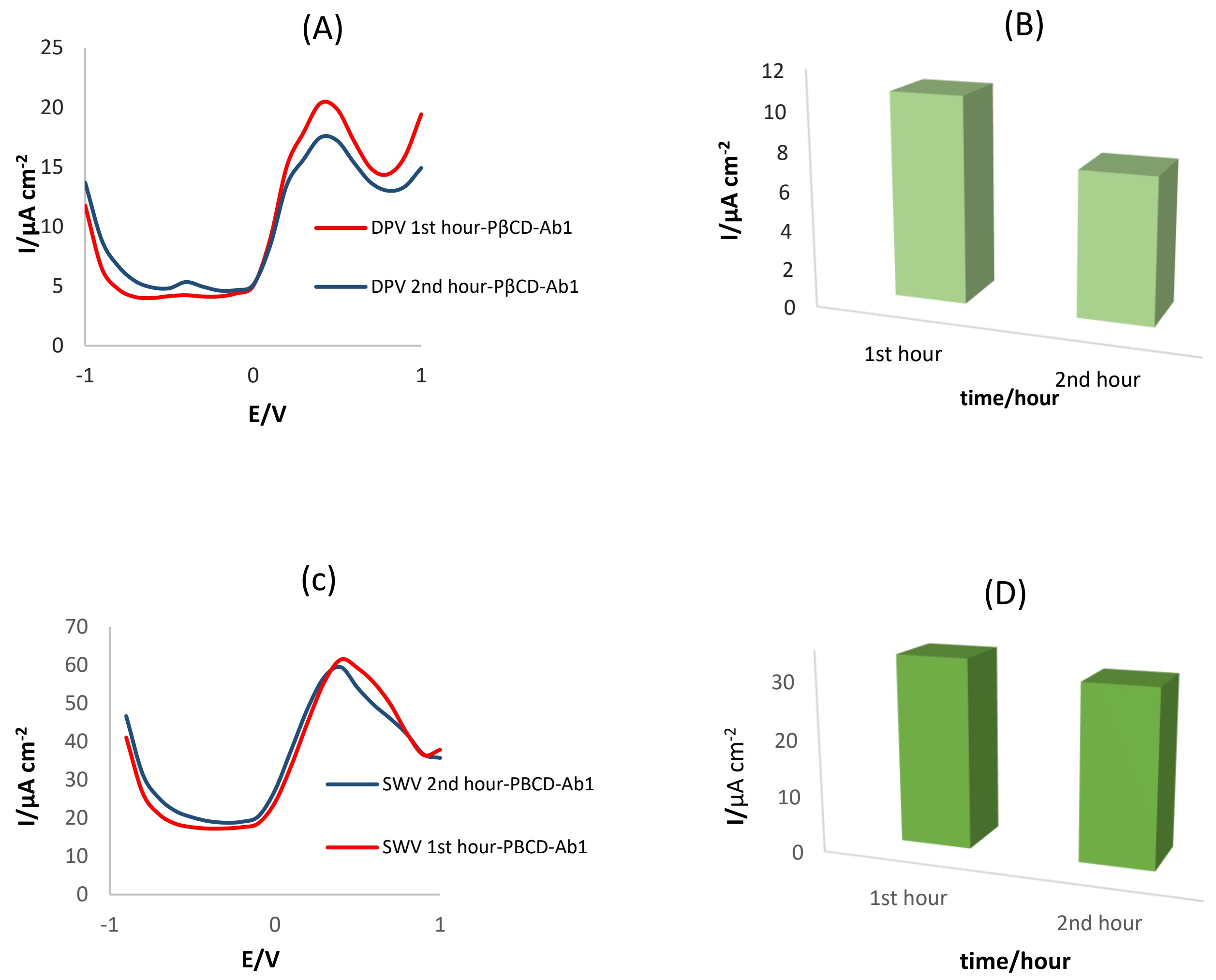

Figure S10. A) DPVs of P(βCD) /Ab1 modified GCE at different time of test. **B)** Histogram of peak current *versus* storage time of test (n=3, SD=1.29). **A)** SWVs of P(βCD) /Ab1 modified GCE at different time of test. **B)** Histogram of peak current *versus* time of test (n=3, SD=2.04).

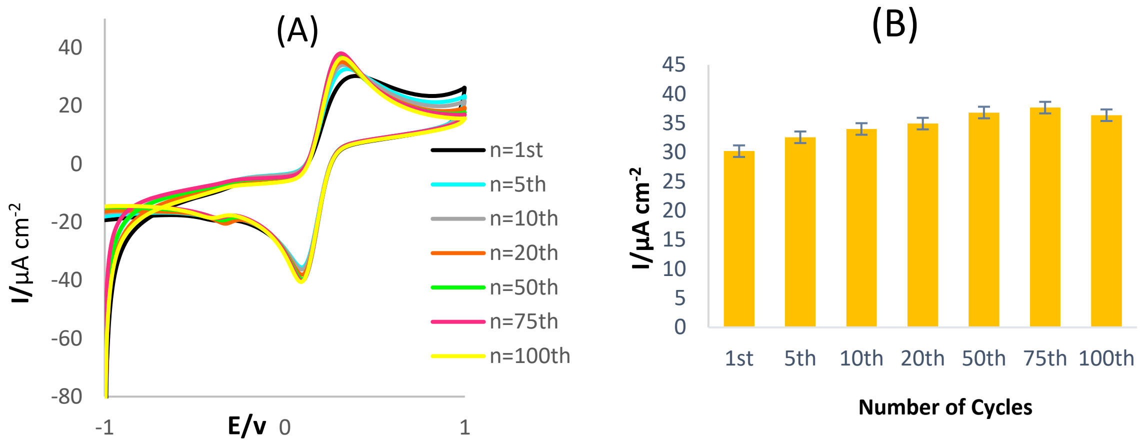

Figure S11. A) CV of P(β-CD)-GCE (polymeric interface) in the potential range of -1 to 1 and scan rate of 0.1 V/s in 0.03M of ferro/ferricyanide in different cycle numbers. **B)** Histogram of peak current *vs* number of cycles (n=3, SD=1.55).

Table S1. Developed analytical methods for detection of 2-AG.

Detection methods	Type of Sample	LOD, LOQ, or LLOQ [a]	Linear range	Limitation/advantages	Ref.
HPLC-MS/MS	Plasma	8.0 ng/mL (LOD)	0.04-10.00 ng mL^{-1}	Expensive, hard to operation	[14]
LC-MS/MS		2 ng mL^{-1} (LOD)	-	Need for advanced tools	[15]
ELISA	Human adipose tissue	5 µM/L(LOD)	5 - 100 µM/L	Expensive, need for advanced process	[16]
LC-MS/MS	Plasma	190 ng mL^{-1} (LOQ)	0.04 - 12.3 ng mL^{-1}	Need for advanced tools	[17]
	Mouse brain	65 fmol (LOQ)	0.02–20 ng mL^{-1}	Expensive, need for expert personal	[18]
Electrochemical immunosensing	Biological	0.00024 ng L^{-1}(LOD)	0.00024–0.0078 ng L^{-1}	Easley and fast operation, no need for advanced tools	[19]
LC-MS/MS	Human cells	1.7 ng mL^{-1} (LOD)	-	Hard to operation, need to expert operator	[20]
Sandwich type electrochemical immunosensing	Human plasma	0.0078 ng/L (LLOQ)	0.0078 - 1.0 ng L	Relatively cheap, easy to operate, fast response	This Study

[a] **LOD**; Limit of detection, **LOQ**; Limit of quantification, **LLOQ**; Low limit of quantification
